# HIV and pre-neoplastic and neoplastic lesions of the cervix in South Africa: a case-control study

**DOI:** 10.1186/1471-2407-6-135

**Published:** 2006-05-23

**Authors:** Jennifer R Moodley, Margaret Hoffman, Henri Carrara, Bruce R Allan, Diane D Cooper, Lynn Rosenberg, Lynette E Denny, Samuel Shapiro, Anna-Lise Williamson

**Affiliations:** 1 School of Public Health and Family Medicine, Women's Health Research Unit, University of Cape Town, 7925, South Africa; 2 Institute of Infectious Disease and Molecular Medicine, University of Cape Town, 7925, South Africa; 3 Slone Epidemiology Unit, Boston University, USA; 4 Department of Obstetrics and Gynaecology, University of Cape Town, 7925, South Africa

## Abstract

**Background:**

Cervical cancer and infection with human immunodeficiency virus (HIV) are both major public health problems in South Africa. The aim of this study was to determine the risk of cervical pre-cancer and cancer among HIV positive women in South Africa.

**Methods:**

Data were derived from a case-control study that examined the association between hormonal contraceptives and invasive cervical cancer. The study was conducted in the Western Cape (South Africa), from January 1998 to December 2001. There were 486 women with invasive cervical cancer, 103 control women with atypical squamous cells of undetermined significance (ASCUS), 53 with low-grade squamous intraepithelial lesions (LSIL), 50 with high-grade squamous intraepithelial lesions (HSIL) and 1159 with normal cytology. Odds ratios (OR) and 95% confidence intervals (CI) were calculated using multiple logistic regression.

**Results:**

The adjusted odds ratios associated with HIV infection were: 4.4 [95% CI (2.3 – 8.4) for ASCUS, 7.4 (3.5 – 15.7) for LSIL, 5.8 (2.4 – 13.6) for HSIL and 1.17 (0.75 – 1.85) for invasive cervical cancer. HIV positive women were nearly 5 times more likely to have high-risk human papillomavirus infection (HR-HPV) present compared to HIV negative women [OR 4.6 (95 % CI 2.8 – 7.5)]. Women infected with both HIV and high-risk HPV had a more than 40 fold higher risk of SIL than women infected with neither of these viruses.

**Conclusion:**

HIV positive women were at an increased risk of cervical pre-cancer, but did not demonstrate an excess risk of invasive cervical cancer. An interaction between HIV and HR-HPV infection was demonstrated. Our findings underscore the importance of developing locally relevant screening and management guidelines for HIV positive women in South Africa.

## Background

Cervical cancer and infection with human immunodeficiency virus (HIV) are both major public health problems in South Africa. The country currently faces one of the worst HIV epidemics in the world. In the past 15 years the epidemic has grown exponentially, with antenatal HIV surveillance data showing an increase in prevalence from less than 1% in 1990 to 29.5 % in 2004 [1]. There are currently more than 5 million people with HIV/AIDS in South Africa, with women more severely affected than men [1].

Cancer of the cervix is the second commonest cancer among South African women, with an age standardized incidence rate of 30 per 100 000 per year [2]. Cervical cancer is included as one of the defining conditions of the acquired immune deficiency syndrome (AIDS) [3]. Studies have shown that HIV positive women are at an increased risk of developing cervical squamous intraepithelial lesions (SIL) and cervical cancer [4-12]. In developed countries it is recommended that HIV positive women be screened for SILs more frequently than HIV negative women [13]. These recommendations are not feasible in developing countries, where both HIV and cervical cancer are common, and resources limited. To develop locally feasible guidelines, data are needed on the prevalence and risk of SILs and cervical cancer among HIV positive women in South Africa. Here we report on one of the first studies that provides information on the risk of SILs among HIV positive women in the Western Cape Province in South Africa.

## Methods

Data were derived from a case-control study that examined the association between hormonal contraceptives and invasive cervical cancer. The study was conducted in the Western Cape from January 1998 to December 2001. Detailed methods have been described elsewhere [14]. There were 524 cases and 1541 controls. Refusal rates were 0.4% and 6.0 %, respectively. Cases were all Coloured and Black women with first occurrences of histologically confirmed invasive cervical cancer, identified at two tertiary gynaecology oncology clinics. The terms Coloured and Black are used to denote groups defined by race classification legislation formerly used in South Africa. In this paper the term Black refers to African people and Coloured to people of mixed racial ancestry.

Nurse interviewers enrolled controls from the local Community Health Centres and the 2 tertiary hospitals. The controls were selected to be representative of the same population from which the cases were identified. They were frequency-matched to the cases for decade of age, ethnic group and area of residence at a ratio of 3:1 controls per case. The trained nurse interviewers collected data on a wide range of variables using a structured questionnaire. Papanicolaou (Pap) smears were taken from the controls and classified according to the Bethesda system [15]. Endocervical scrapings were taken from the controls only and were assayed for human papillomavirus (HPV) infections using the Hybrid Capture II HPV test for the detection of high-risk HPV (HR-HPV) types. The cervical cancer cases were considered positive for HR-HPV. At the time of the study serum samples were taken and stored.

The study was later extended to explore the relationship between HIV and cervical pre-cancer and cancer. Anonymous HIV testing was done on stored serum samples, using the enzyme-linked immunoabsorbant assay (ELISA). HIV data were available for 486 (93%) of the 524 cases of cervical cancer, and HIV and Pap results for 1365 (89%) of the original controls.

For the purposes of the present analysis we divided the patients into several groups. There were 486 women with invasive cervical cancer, 103 control women had atypical squamous cells of undetermined significance (ASCUS), 53 had low-grade squamous intraepithelial lesions (LSIL), 50 had high-grade squamous intraepithelial lesions (HSIL) and 1159 had normal cytology.

Written informed consent for the overall study was obtained from all study participants. Ethical approval for the overall study and, later, for the anonymous HIV testing was granted by the University of Cape Town Research Ethics Committee and the Boston University Ethics Review Board.

Socio-demographic and reproductive characteristics were examined using chi square and Kruskal-Wallis tests. The median ages of HIV positive and HIV negative women were compared using the Wilcoxan sign rank test. For the association between HIV and invasive cervical cancer we compared HIV prevalence in the cases with that in all controls. For the association between HIV and SIL, women with normal cytology were used as a reference group. Statistical analysis was carried out using unconditional multiple logistic regression (SAS version 9.1). Initial models contained multiple variables that were considered to be potential confounders (age, ethnic group, number of sexual partners, education level, urban or rural residence, marital status, presence of sexually transmitted infection, number of prior Pap smears, parity, age at coitarche, alcohol use, tobacco use, sterilization, use of oral contraceptives, and use of injectable contraceptives). A backward stepwise regression was performed. Variables were retained in the model if they demonstrated independent associations with the outcome of interest, or if their removal altered the association between HIV infections and the outcome of interest. Systematic elimination of variables resulted in odds ratio (OR) estimates adjusted for age and ethnic group.

An additional analysis, which took into account joint HIV/HR-HPV status was conducted. In this analysis women were stratified women according to whether they were HR-HPV positive or negative and HIV positive or negative. In this analysis we controlled for age and ethnicity.

## Results

### Socio-demographic and reproductive characteristics

Table 1 provides socio-demographic and reproductive characteristic for the cases and controls for whom HIV data was available. Cases of invasive cervical cancer were more likely to have 4 or more sexual partners, had their sexual debut before the age of 16 years, and smoked cigarettes, and were less likely to have used hormonal contraceptive and have had a prior Pap smear.

### HIV and invasive cervical cancer

Overall 5.7% (78) of the HIV tested controls (1365) and 6.0% (29) of the tested cases (486) were HIV positive. The odds ratio for the association between HIV and invasive cervical cancer was 1.17 (95% CI 0.75 – 1.85), estimates adjusted for age and ethnicity.

Among women with cervical cancer, those that were HIV positive were 6 years younger that those that were HIV negative (median age 40 years and 46 years for HIV positive and HIV negative women respectively, p = 0.004).

Overall, the majority of women with cervical cancer presented with late stage disease (stages III and IV). Analysis of the prevalence of HIV infection according to stage of cancer showed that 8.7% (12/138) of stage Ib and II were HIV infected compared to 4.9% (17/346) of stage III and IV cancers (p = 0.17).

### HIV and SIL

Among the controls, 50% (39/78) of HIV positive women had an abnormal Pap smear (20.5% ASCUS, 17.9% LSIL and 11.5% HSIL), and 13% of the HIV negative controls (167/1287) had an abnormal Pap smear (6.8% ASCUS, 3.0% LSIL and 3.2% HSIL). Table 2 gives the distribution of HIV positivity in each of the groups according to cytology. With normal women as the reference category the adjusted odds ratios associated with HIV infection were: ASCUS 4.4 (95% CI 2.3 – 8.4), LSIL 7.4 (95% CI 3.5 – 15.7) and HSIL 5.8 (95% CI 2.4 – 13.6).

Among women with pre-cancerous lesions, HIV positive women tended to be younger, but the age differences were not statistically significant for ASCUS or HSIL and only of borderline significance for LSIL (p = 0.05).

### HIV, high-risk HPV and cervical lesions

Among the controls 49% (38/78) of the HIV positive women were positive for HR-HPV, compared to 15% (193/1286) of the HIV negative women. HIV positive women were almost 5 times more likely to have HR-HPV present compared to HIV negative women [OR 4.6 (95 % CI 2.8–7.5)]. Women infected with HIV and HR-HPV were at a higher risk of SIL than women infected with neither of these viruses [OR 41.3; 95% CI (18.8–90.5)] (Table 3).

## Discussion

This study provides data on the risk of cervical SILs and invasive cervical cancer among HIV positive women less than 60 years old in the Western Cape Province in South Africa. The extent to which HIV increases the risk for cervical cancer is especially important in South Africa given the size of the HIV epidemic. Studies have produced inconsistent results on the association between HIV and invasive cervical cancer [10-12]. In our study HIV positive women did not have an excess risk of invasive cervical cancer. This could be due to the competing risk of mortality from other conditions associated with HIV, particularly in a setting where antiretroviral therapy was not available at the time.

Similar to other studies conducted in South Africa, the majority of women in our study, irrespective of HIV status presented with late stage disease [16]. We found no association between HIV and the stage of cervical cancer. HIV positive women with invasive cervical cancer were 6 years younger than HIV negative women. This difference in mean age between HIV positive and negative women is difference in mean age is smaller than that reported in other South African studies [16,17] and probably reflects the earlier stage of the HIV epidemic in the Western Cape compared to the other study sites.

Our finding that HIV infected women were at a significantly higher risk of LSIL and HSIL confirms the findings of studies from both developed and developing countries [4-9,11]. The prevalence (50%) of cytological abnormalities among HIV positive women is higher than has been reported by any other study in Africa. Hawes et al reported a cytological abnormality prevalence of 37% among women with HIV-1 infection attending an outpatient infectious-disease clinic in Senegal [6], Chirenje 26 % among family planning attendants in Zimbabwe [7], Laga 27% in sex workers in Zaire [18], Maggwa 5% among family planning attendants in Kenya [19] and Kreiss 26% among sex workers in Kenya [20]. The controls in the case-control study (from which our subjects were derived) were frequency matched for age to cases of invasive cervical cancer, therefore, our study consists of slightly older women than those in the above mentioned studies. This difference in age could account in part for the higher prevalence of cervical abnormality recorded in our study.

A potential limitation of our study is that cytological abnormalities were not histologically confirmed. However, it has been shown that the Pap smear sensitivity and specificity are similar among HIV negative and HIV positive women [4,21]. Further, the cytologists were blinded to HIV status and there is no reason to suspect differential misclassification according to HIV status. Random misclassification would dilute the estimated effect of HIV. An additional limitation of our study was that immune status was not recorded. A strong association between immune status (CD4 counts and HIV viral load) and cervical abnormalities has been demonstrated in other studies [5,8,9].

In developed countries it is recommended that HIV positive women have two cytological assessments within the first year after HIV diagnosis and annually thereafter, with referral for colposcopy for any smear showing an ASCUS or more severe lesion [13]. These guidelines are not feasible in resource-poor settings. The high prevalence and risk of cervical abnormalities documented in our study underscores the importance of developing screening and management guidelines for HIV positive women. As anti-retroviral therapy becomes increasingly available in the public sector in South Africa, the life expectancy of HIV positive women will increase. It is important that this benefit is not offset by an excess risk in cervical cancer. It is of critical importance that a cervical screening program, informed by local research on the natural history of cervical abnormalities, is in place for HIV positive women.

South Africa has recently introduced a national cervical screening policy that all women 30 years and older are entitled to three free Pap smears in their lifetime, at 10 year intervals [22]. Over the next few years many women in South Africa will be screened for the first time for cervical abnormalities. According to the policy all women with LSIL should have a repeat smear in 1 year, and all women with HSIL referred for a colposcopy assessment. Based on our study findings that 1 in 4 women with LSIL, and 1 in 5 women with HSIL, were HIV positive, we recommend that every effort should be made to ensure that women who are screened and found to have SILs should also be offered HIV voluntary counselling and testing.

Our results are in agreement with others that have shown an association between HIV, high-risk HPV and cervical abnormalities [8,23]. For the combination of HIV and HPV infection, we observed an interaction with an estimated 41-fold increase in the risk of LSIL and HSIL combined. This striking increase underscores the need to study the mechanisms underlying the interaction in the pathogenesis of cervical SIL.

## Conclusion

In summary South African HIV positive women are at a significantly increased risk of cervical HR-HPV infection and SILs. The increased public health burden that this poses is an important and gender-specific aspect of HIV infection. Guidelines on how best to screen HIV positive women for cervical abnormalities are urgently needed in South Africa.

## Competing interests

The author(s) declare that they have no competing interests.

## Authors' contributions

JM was involved in designing the study and in the data analysis, and drafted the manuscript.

MH was involved in proposal writing, supervision of field staff and data analysis.

HC was responsible for detailed data analysis.

BA carried out the virology assessments.

DC supervised the field work.

LR was involved in proposal writing.

LD was the clinical consultant on the project.

SS was involved in proposal writing and data analysis.

AW was the principal investigator and was responsible for virological assessments including laboratory based tests.

All authors and read and approved the final manuscript.

## Pre-publication history

The pre-publication history for this paper can be accessed here:



## Figures and Tables

**Table 1:** Socio-demographic and reproductive characteristics

**Characteristic**	**Cases (N = 486)**	**Controls (N = 1 365)**	P value*
Age			
< 35 years	61 (12.5)	219 (16.0)	
≥ 35 years	425 (87.5)	1 146 (84.0)	0.065
Ethnic group			
Coloured	367 (75.5)	1 030 (75.5)	
Black	119 (24.5)	335 (24.4)	0.980
Residence			
Rural	208 (42.8)	632 (46.3)	
Urban	278 (57.2)	733 (53.7)	0.183
Marital status			
Single	116 (23.9)	297 (21.8)	
Married	223 (45.9)	704 (51.6)	
Widowed	70 (14.4)	158 (11.6)	
Divorced/separated	76 (15.6)	205 (15.0)	0.197
Sexual partners			
< 4	355 (73.1)	1 105 (81.0)	
≥ 4	127 (26.1)	252 (18.4)	
Missing	4 (0.8)	8 (0.6)	< 0.001
Sexual debut			
< 16 years	156 (32.1)	319 (23.4)	
≥ 16 years	318 (65.4)	1 008 (73.8)	
Missing	12 (2.5)	38 (2.8)	< 0.001
Hormonal contraceptive use			
Never	117 (24.1)	197 (14.4)	
Ever	369 (75.9)	1 168 (85.6)	< 0.001
Sterilized			
No	361 (74.3)	859 (62.9)	
Yes	125 (25.7)	506 (37.1)	< 0.001
Tobacco use			
Never	159 (32.9)	550 (40.3)	
Ever	326 (67.1)	815 (59.7)	0.004
Prior Pap smear			
No	242 (49.8)	370 (27.1)	
Yes	238 (49.0)	982 (71.9)	
Missing	6 (1.2)	13 (1.0)	< 0.001

* Excludes missing category

**Table 2:** HIV status and risk of SIL

	**N**	**HIV positive N (%)**	**HIV negative N (%)**	Adjusted OR* (95% CI)
Normal	1 159	39 (3.0)	1 120 (97.0)	1.0 (reference)
ASCUS	103	16 (15.5)	87 (84.5)	4.4 (2.3 – 8.4)
LSIL	53	14 (26.4)	39 (73.6)	7.4 (3.5 – 15.7)
HSIL	50	9 (18.0)	41 (82.0)	5.8 (2.4 – 13.6)

* OR adjusted for age and ethnicity

**Table 3:** HIV, HR-HPV and risk of SIL

	**SIL absent N**	**SIL present N (%)**	Adjusted OR* (95% CI)
HIV – HR-HPV-	1 070	23 (2.1 %))	Reference
HIV+ HR-HPV-	35	5 (12.5 %)	6.5 (2.3 – 33.0)
HIV- HR-HPV+	136	57 (29.5 %)	19.6 (11.7 – 33.0)
HIV+ HR-HPV+	20	18 (47.4 %)	41.3 (18.8 – 90.5)

* OR adjusted for age and ethnicity

SIL absent includes Paps that were normal and had ASCUS. SIL present include LSIL and HSIL Paps.
